# Modern Endophthalmitis Control: The Complete Early Vitrectomy for Endophthalmitis (CEVE) Protocol and Surgical Prophylaxis

**DOI:** 10.7759/cureus.91513

**Published:** 2025-09-03

**Authors:** Agnieszka J Kudasiewicz-Kardaszewska, Malgorzata A Ozimek, Aleksandra Kardaszewska, Karolina Boninska, Ferenc Kuhn, Slawomir Cisiecki

**Affiliations:** 1 Ophthalmology, Ośrodek Chirurgii Oka Prof. Zagorskiego sp. zoo w Nowym Sączu, Grupa Medyczna OCHO, Nowy Sącz, POL; 2 Dentistry, Uniwersyteckie Centrum Stomatologii, Medical University of Lublin, Lublin, POL; 3 Ophthalmology, Miejskie Centrum Medyczne im. dr. Karola Jonschera w Łodzi, Łódź, POL; 4 Ophthalmology, Helen Keller Foundation for Research and Education, Birmingham, USA; 5 Ophthalmology, University of Pécs, Pécs, HUN

**Keywords:** antibiotics, endophthalmitis, management algorithm, ocular surgery, povidone-iodine, prevention, vitrectomy

## Abstract

This article presents an overview of endophthalmitis, a severe intraocular inflammatory condition that can develop following surgery, intravitreal injections (IVIs), or ocular trauma. Endogenous cases may also arise, particularly in patients with compromised immune systems. A broad spectrum of pathogens, including bacteria, fungi, and viruses, can be involved, with some displaying high virulence. Given the rapid progression and potential for vision-threatening complications or systemic involvement such as sepsis, immediate and appropriate intervention is essential. Surgical management, especially vitrectomy, plays a central role in treatment.

The Complete Early Vitrectomy for Endophthalmitis (CEVE) protocol has demonstrated high effectiveness, particularly when integrated into outpatient ophthalmic surgical practices. This review describes the protocol's practical application in such settings. In addition, it discusses preventive strategies for ocular surgery and injections, with a particular focus on the judicious use of antimicrobial agents. Incorrect antibiotic use, including unnecessary prescribing, inadequate dosing, or prolonged treatment, remains a leading contributor to the development of antimicrobial resistance and warrants critical evaluation.

In summary, the CEVE approach appears to offer broad efficacy across different causes of endophthalmitis. Povidone-iodine (PI) remains the most effective agent for perioperative antisepsis. The use of prophylactic antibiotics should be confined to brief, high-potency topical therapy following intraocular procedures, including cataract surgery and phacovitrectomy. Current evidence does not support the routine use of prophylactic antibiotics after intravitreal injections due to minimal benefit and the risk of promoting resistant organisms.

## Introduction and background

Endophthalmitis is a severe intraocular infection involving the inflammation of the ocular fluids and tissues. It is often characterized by the accumulation of pus in the vitreous cavity and the infiltration of the inner eye wall [1,2]. The condition may arise after ocular surgery, intravitreal injections (IVIs), and trauma or through endogenous spread. Although bacteria are the most common pathogens, fungi and viruses can also be responsible. It constitutes an ophthalmic emergency, with potential for rapid, irreversible vision loss or even enucleation or evisceration. Acute postoperative endophthalmitis typically presents within 1-4 days postoperatively, although onset may occur up to two weeks later. For this reason, it is of utmost importance to intervene immediately and perform Complete Early Vitrectomy for Endophthalmitis (CEVE). This protocol, introduced in 2008, is based on the historical surgical principle "ubi pus, ibi evacua," which means "where there is pus, evacuate it" [1,2].

Endophthalmitis requires immediate treatment. From both medical and legal perspectives, therapy should be initiated without delay, regardless of the chosen approach. The presence of pus inside the eye necessitates urgent evacuation, as it can rapidly lead to irreversible vision loss [1-3]. Therefore, we advocate for immediate surgical management, accompanied by the systemic administration of antibiotics such as vancomycin, cephalosporins, or fluoroquinolones [4,5].

Complete early vitrectomy is essential for retinal survival, particularly given that the causative organism is usually unknown at presentation and the infection may progress rapidly [1]. Since most ocular surgeries are now performed on an outpatient basis, we propose a protocol for managing endophthalmitis in one-day surgery centers, ensuring timely and optimal intervention.

## Review

Methods of literature search

A structured literature search was performed in PubMed, Scopus, and Web of Science databases. Articles published between 1995 and 2025 were considered, with particular emphasis on the most recent decade to capture contemporary prophylactic and surgical practices. Inclusion criteria comprised peer-reviewed clinical trials, cohort studies, large case series, systematic reviews, and guidelines focused on the management or prevention of endophthalmitis, including vitrectomy approaches (notably CEVE), antibiotic prophylaxis, and perioperative antiseptic measures. We excluded non-peer-reviewed publications, single case reports with fewer than five patients (unless reporting unique complications or CEVE applications), non-English articles without available translation, and duplicate entries across databases. In total, 50 articles were reviewed in detail, including 12 multicenter studies, eight systematic reviews or meta-analyses, and 30 original research articles or large case series. References were cross-checked manually to ensure comprehensive coverage.

Aim and scope

The aim of this narrative review is to provide a structured and practical summary of endophthalmitis management, with particular emphasis on the Complete Early Vitrectomy for Endophthalmitis (CEVE) protocol. The proposed algorithm is tailored specifically for one-day eye surgery centers, ensuring it is both applicable and feasible in outpatient surgical settings.

Endophthalmitis management in a one-day eye surgery clinic

**Figure 1:**
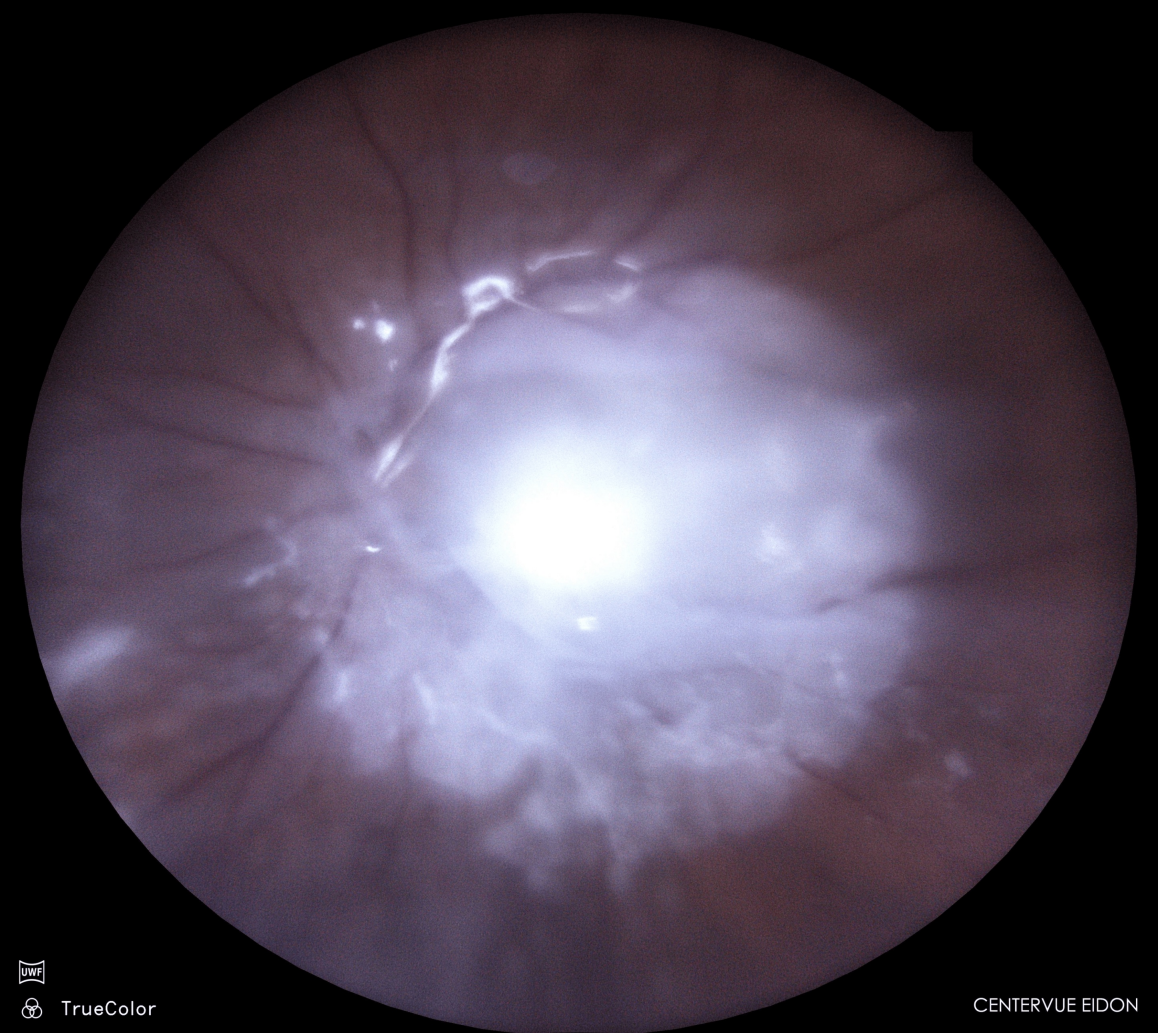
Acute postoperative endophthalmitis (preoperative photograph) Note the thick "blanket" of pus covering the macula. Preoperative picture taken by Małgorzata Ozimek, MD, PhD, in Prof. Zagorski Eye Surgery Centre in Nowy Sącz (Eidon FA, CenterVue, Padua, Italy)

Endophthalmitis (Figure 1) is a rare but potentially devastating condition that requires immediate and effective intervention to preserve the patient's vision and ocular structures and, in cases involving highly virulent pathogens, even the patient's life [1,3]. The prompt diagnosis and swift initiation of treatment are essential to avoid irreversible damage and optimize visual outcomes in this critical clinical scenario.

**Figure 2:**
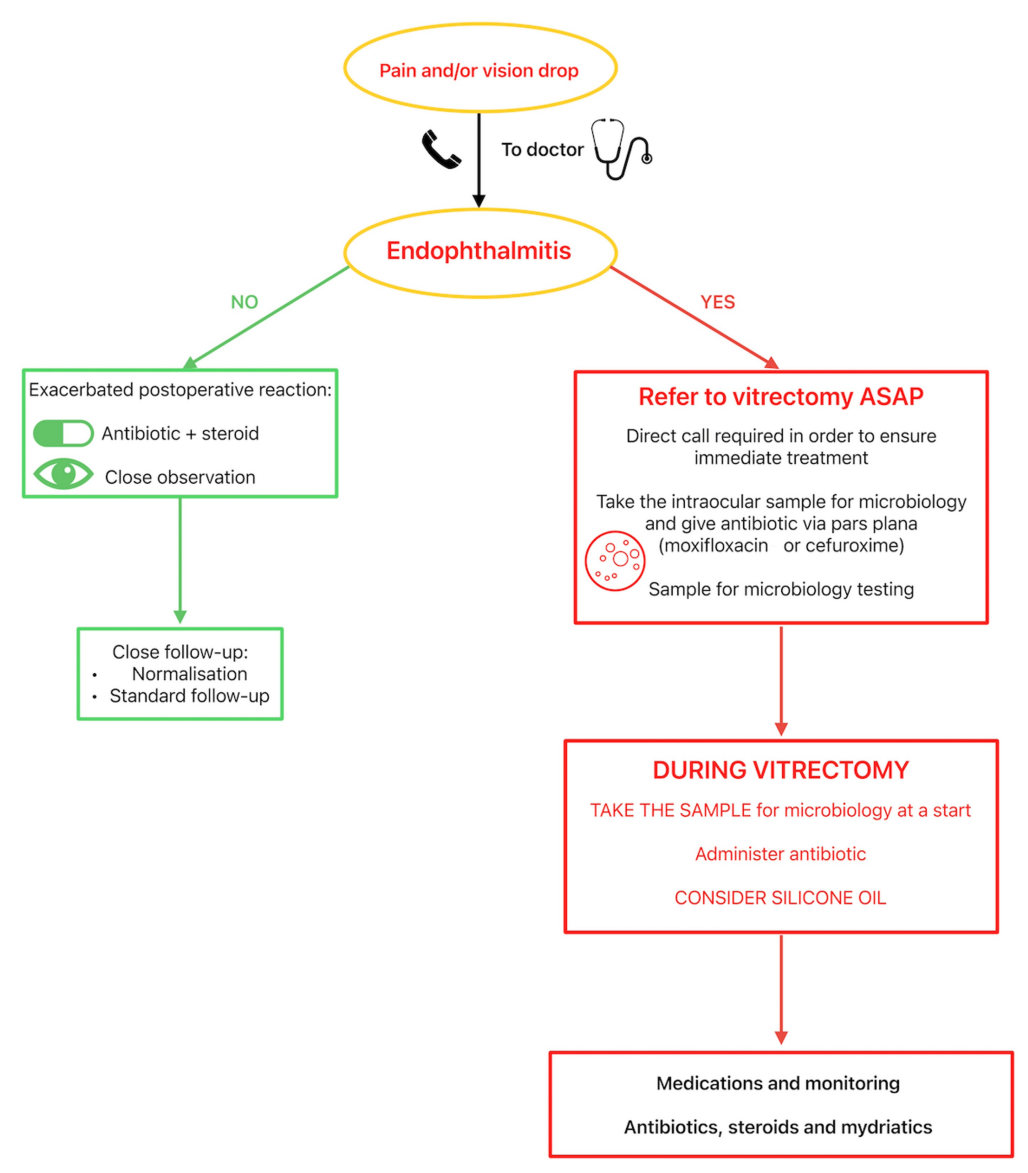
Endophthalmitis management in a one-day clinic setup The image was created by Aleksandra Kardaszewska, MD, based on a decision-making tree in the standard operating procedure of endophthalmitis in the OCHO Medical Group

Every patient experiencing pain and/or vision deterioration after an operation or injection who contacts the registration office of the clinic should be examined as soon as possible, no later than on the same day. If the physician detects symptoms or suspects endophthalmitis, the patient should be urgently referred to a vitreoretinal (VR) surgeon. It is advisable to have direct contact with a VR surgeon affiliated with the clinic or to arrange an immediate referral to a hospital equipped with a VR surgery unit. A sample for microbiological testing should be obtained either through a pars plana tap before referring the patient to a VR surgeon or at the beginning of vitrectomy [6-8]. If the patient must travel a significant distance to the VR surgery unit, it is advisable to take a pars plana tap before referral and administer an intravitreal antibiotic injection (e.g., moxifloxacin, levofloxacin, or cephalosporin) via pars plana. This may help control the infection until a proper vitrectomy can be performed. We recommend implementing the Complete Early Vitrectomy for Endophthalmitis (CEVE) strategy, which has been proven to be the most effective approach for endophthalmitis management. Figure 2 describes the endophthalmitis management algorithm in a one-day surgery clinic.

**Figure 3:**
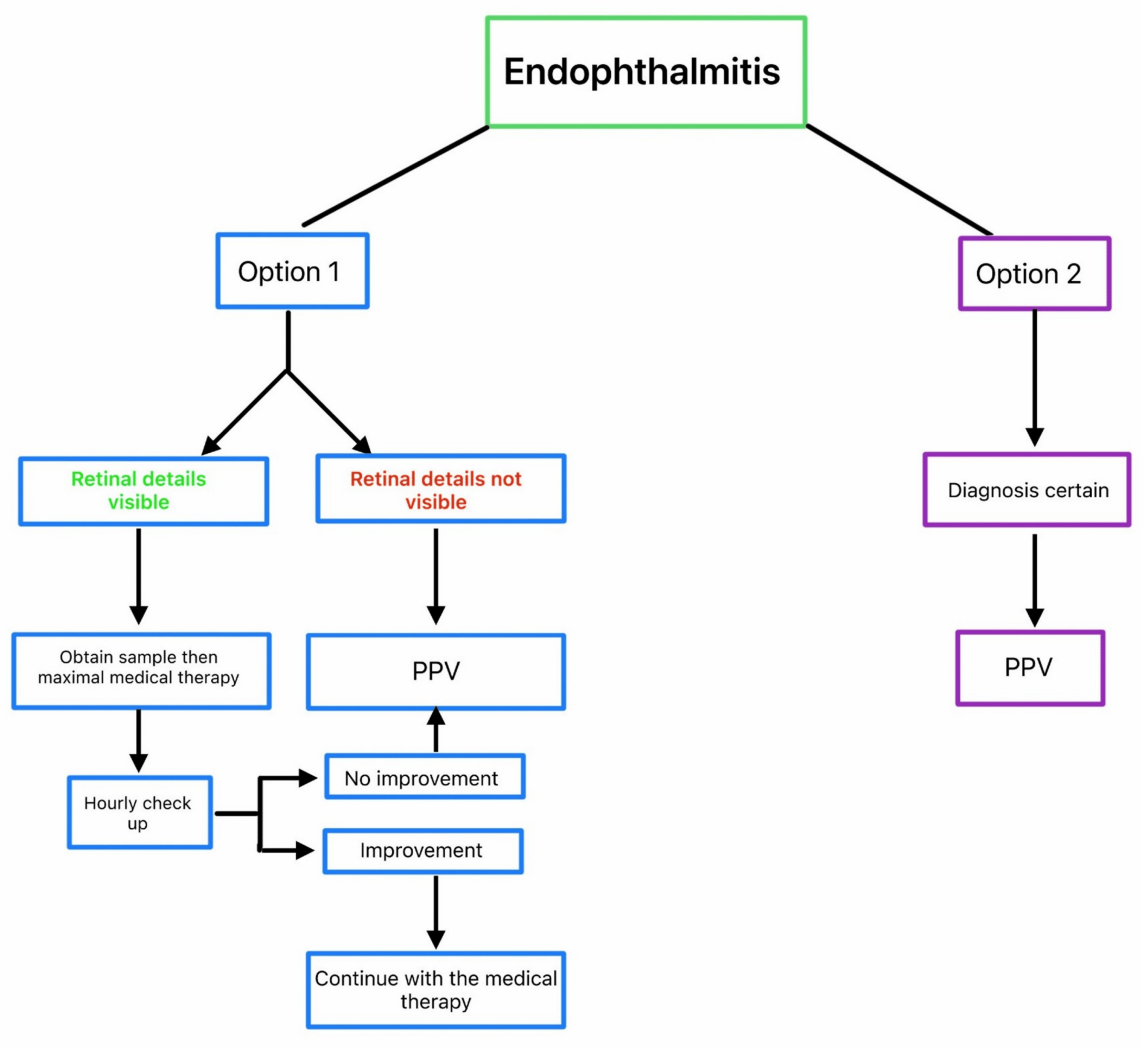
Decision-making tree for endophthalmitis In both options, vitrectomy seemed to be the default strategy in efficient endophthalmitis management. A schematic diagram was created by Aleksandra Kardaszewska, adapted from Kuhn [1], and reviewed and consulted with Ferenc Kuhn, the author of the concept PPV: pars plana vitrectomy

The algorithm (Figure 2) provides a straightforward guideline for practicing ophthalmologists. If there is any doubt, the condition should be treated as endophthalmitis, and vitrectomy should be performed as soon as possible. The presence of pus inside the eye requires its immediate evacuation and the use of all available treatment modalities against virulent pathogens [1,5]. For this reason, pars plana vitrectomy (PPV) is the treatment of choice (Figure 3, depicted as option 2). However, if the retina is visible, a sample may be taken via a pars plana tap, and the patient can be placed on intensive medical therapy with close follow-up (Figure 3, option 1).

Pars plana vitrectomy (PPV) for endophthalmitis should have several key steps [9,10]. After pars plana trocar insertion, the surgeon should obtain samples from the vitreous cavity and the anterior chamber for microbiological testing. During vitrectomy, a broad-spectrum antibiotic via infusion fluid (e.g., vancomycin 0.2 mg/mL in 500 mL Ringer's solution) should be administered [4,5]. Intraoperative anterior chamber lavage is performed using a broad-spectrum antibiotic. Pus is removed with the vitrector, ensuring posterior vitreous detachment to clean the retinal surface as thoroughly as possible [1]. The ciliary body and the vitreous base should be examined with indentation. Any preexisting ocular pathologies, such as retinal detachment, must be addressed [1]. At the end of the procedure, an appropriate intravitreal antibiotic should be left in the vitreous cavity (see Table 1) [6].

**Table 1:** Intravitreal antibiotics and their doses Note: Reconstitute antibiotics with the included solvent and dilute with Ringer's solution or normal saline. Specific names for medications are indicated in parentheses. The table was created by Agnieszka Kudasiewicz-Kardaszewska, MD, PhD, with the use of the data extracted from Pietras-Baczewska et al. [4], Althiabi et al. [6], Brockhaus et al. [9], and Weber et al. [10]

Antibiotic agent	Dose	References
Moxifloxacin (Vigamox)	500 µg/0.1 mL	[9]
Vancomycin (Vancocin)	1 mg/0.1 mL	[4]
Ceftazidime (e.g., Biotum)	2.2 mg/0.1 mL	[6]
Cefuroxime (Aprokam)	1 mg/0.1 mL	[9]
Amikacin (Amikin)	0.4 mg/0.1 mL	[9]
Amphotericin B (Fungizone), antifungal agent	5 µg/0.1 mL	[9,10]

Silicone oil tamponade should be considered, as silicone oil has bacteriostatic properties and may help prevent late complications such as retinal detachment or eyeball atrophy [10,11].

**Figure 4:**
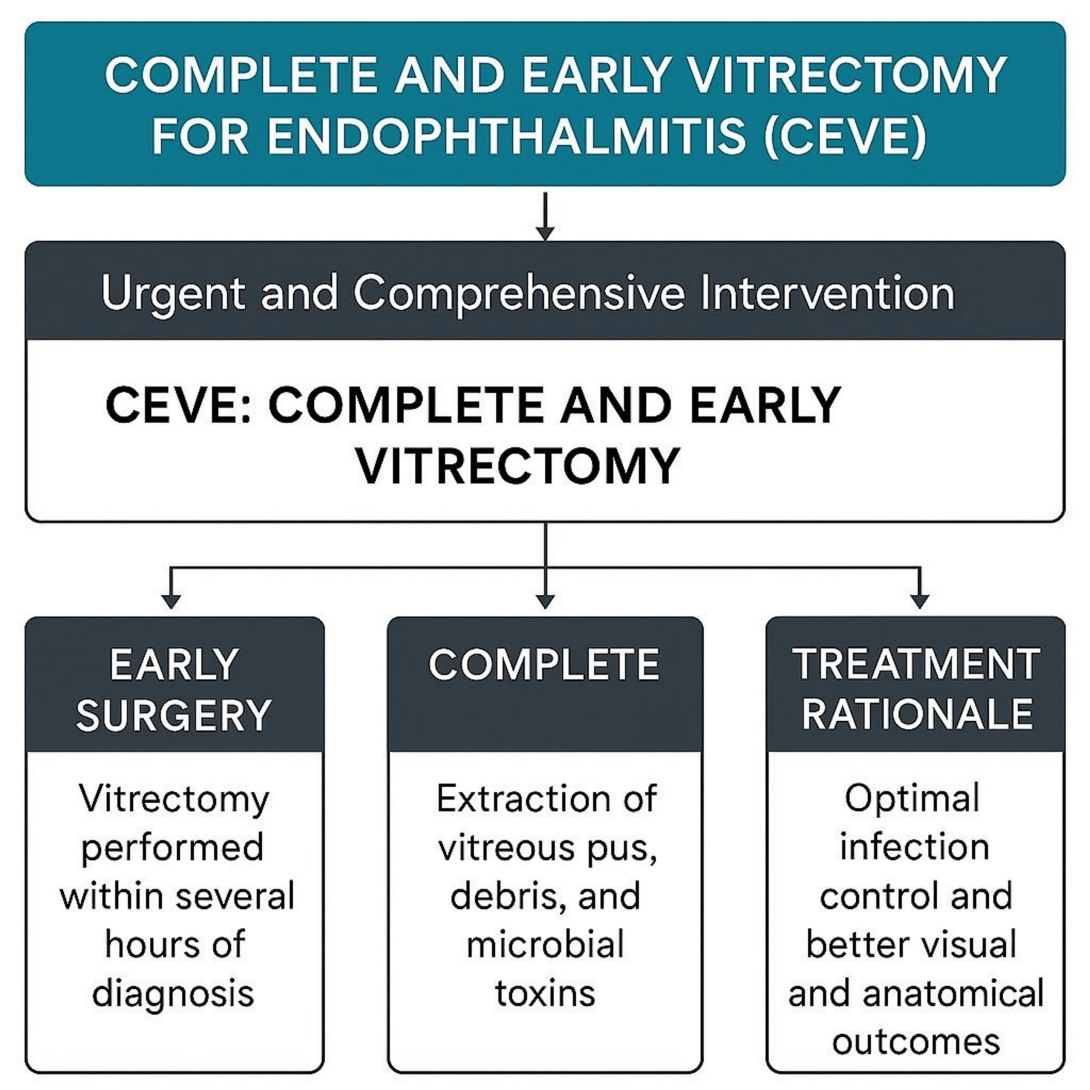
Diagram representing CEVE definition Diagram made by Agnieszka Kudasiewicz-Kardaszewska, MD, PhD, with the use of Canva AI (Canva, Sydney, Australia)

The CEVE protocol mandates immediate surgical intervention following diagnosis, ideally before significant intraocular tissue damage has occurred. The term "early" refers to this critical window, during which prompt action can preserve retinal function [2]. The procedure must be as thorough as possible to remove all bacterial organisms, toxins, and inflammatory debris from the vitreous cavity (Figure 4). A key component of CEVE is the removal of pus from the posterior retina, commonly referred to as "macular hypopyon," which requires the detachment of the posterior hyaloid [1]. This must be done carefully to avoid macular damage. Although termed "complete," the vitrectomy is intentionally limited in the anterior segment, specifically at the vitreous base, to reduce the risk of iatrogenic retinal tears, which may lead to postoperative detachment [12,13].

Following vitrectomy, intensive topical therapy is required. Broad-spectrum antibiotics should be administered hourly, accompanied by topical corticosteroids such as dexamethasone every 2-3 hours. Mydriatic agents (e.g., atropine, cyclopentolate, or tropicamide) should be instilled 2-3 times daily to prevent posterior synechiae and ease discomfort [14,15]. Treatment must be adjusted once culture and sensitivity (antibiogram) results are available. If fortified antibiotic drops are indicated, they should be prepared by a hospital pharmacy under sterile conditions [16]. Every case of endophthalmitis should be reported to the infection control group within the clinic or hospital and documented in the Electronic Adverse Events Registry.

While CEVE forms the cornerstone of our recommended management approach, it must be evaluated in the context of alternative strategies historically used to treat endophthalmitis [1,2,4,17]. The following section compares CEVE with other protocols, including the Endophthalmitis Vitrectomy Study (EVS)-guided model, tap and inject, and delayed vitrectomy [3,18-20]. Each approach has distinct advantages and limitations, which are summarized in Table 2.

**Table 2:** Alternative approaches to endophthalmitis management with their pros and cons The table was created by Agnieszka Kudasiewicz-Kardaszewska, MD, PhD EVS: Endophthalmitis Vitrectomy Study

Approach	Description	Advantages	Limitations	References
Tap and inject	Vitreous sampling, followed by intravitreal antibiotics	Rapid, minimally invasive	No removal of infectious material; suboptimal in aggressive infections	[3]
Delayed vitrectomy	Initial antibiotic therapy, with surgery only if the condition worsens	Avoids surgical intervention if improvement occurs	Delays pus removal; may increase the risk of retinal complications	[18]
EVS-guided strategy	Vitrectomy limited to cases with light perception vision or worse	Historically evidence-based; conservative approach	Based on outdated technology; may miss the benefits of early intervention	[20]

Historically, management strategies for acute postoperative endophthalmitis have ranged from conservative antibiotic-based approaches to delayed surgical intervention. The Endophthalmitis Vitrectomy Study (EVS) remained one of the most influential trials in this domain, recommending vitrectomy only for patients with visual acuity of light perception (LP) or worse [20]. However, advances in surgical techniques and instrumentation have led to a paradigm shift favoring earlier vitrectomy. Recent multicenter studies (e.g., European Vitreo-Retinal Society Endophthalmitis Study [15]) and case series have supported the superiority of CEVE in improving anatomic and functional outcomes, especially in fulminant or uncertain cases [5,10,17]. CEVE facilitates pathogen sampling, reduces microbial load, and enables the concurrent management of complications (e.g., retinal detachment or vitreous hemorrhage) [15,17]. With advancements in minimally invasive vitrectomy techniques (e.g., 23G and 25G systems), CEVE is now feasible even in outpatient settings. In this context, it represents a modern proactive strategy for managing a condition in which delayed intervention often leads to irreversible outcomes [15,17,19].

Prevent before it happens

The prevention of endophthalmitis during ophthalmic surgery, including cataract, vitreoretinal, or combined procedures, requires a multistage protocol incorporating preoperative, intraoperative, and postoperative components. Prior to surgery, the eyelid margins should be evaluated for conditions such as blepharitis, meibomian gland dysfunction, rosacea, or excessive biofilm on the eyelashes [21]. These conditions must be addressed in advance to rebalance the ocular surface and reduce the bacterial load, as many postoperative infections originate from the patient's own flora. Organisms such as *Staphylococcus epidermidis* and *Staphylococcus aureus*,* *both common components of normal conjunctival flora, can become pathogenic when introduced into the intraocular space [21]. While complete sterilization is impossible, effective decontamination and surface balance are achievable [22]. Minimum recommended treatments for chronic blepharitis include eyelid thermotherapy, lid hygiene with special wipes, and preservative-free artificial tears [21]. In severe cases, mupirocin ointment may be applied to the nasal passages and periorbital skin to reduce *Staphylococcus* colonization, including methicillin-resistant *Staphylococcus aureus* (MRSA). A suggested regimen includes applying mupirocin to both nostrils twice daily for five days before surgery [23].

Prevention in the operating room (OR)

All personnel present in the OR should wear proper attire, including gowns, caps, boots, and masks covering both the mouth and nose [22]. If a mask becomes wet or contaminated, it must be replaced immediately. Airflow systems, such as laminar flow or high-efficiency particulate air (HEPA)-filtered air-conditioning, should be operational at least one hour prior to the start of surgery and remain active throughout the procedure. Turning off the airflow system is discouraged. Surgeons who experience discomfort may wear an additional layer beneath the gown. The surgeon must carefully scrub their hands using sterile soap and warm water, paying special attention to nail hygiene. Nails should be short and free of polish or debris. Special sterile spatulas can be used to clean under the nails. Handwashing should last at least 90 seconds up to three minutes, depending on the detergent soap used [24]. According to the WHO (2008), if antimicrobial soap and clean water are available, additional disinfection is not necessary [24]. However, in practical settings, surgeons should perform surgical handwashing to remove any visible contaminants before entering the OR. Subsequently, a disinfectant should be applied to the hands and forearms for at least 1-3 minutes (up to five minutes if necessary), depending on the agent used. Disinfection targets the surgeon's and nurses' own flora and should be repeated between surgeries. Handwashing should also be performed after using the restroom, eating, encountering blood or body fluids, or returning to the OR from another area [25]. After hand scrubbing and disinfection, the surgical team should wear a sterile gown and gloves. The circulating nurse should prepare a table with sterilized and disposable sterile equipment. Either the circulating nurse or the surgeon should disinfect the operative field. This involves applying 7.5% or 10% povidone-iodine (PI) to the eyelids, orbital area, forehead, and cheek (twice for 30 seconds each time) and 5% PI to the ocular surface before surgery and after speculum insertion, 30 seconds each time. PI has been proven to be the most effective antiseptic for preventing postoperative endophthalmitis [22,26]. PI, a broad-spectrum antimicrobial agent, works by destroying microbial DNA and proteins [27]. It is effective against bacteria, viruses, chlamydia, and fungi. Evidence shows that it can reduce the ocular surface bacterial load by up to 100-fold [22,26].

Controversies regarding preoperative topical antibiotics

There is ongoing debate regarding the use of topical antibiotics (e.g., levofloxacin or tobramycin) before surgery. Based on our experience, it may be beneficial, especially for patients with stage III or IV blepharitis (even in remission) or monocular patients [21]. However, antibiotics should be instilled at least 30 minutes before surgery. Some surgeons advocate for administering antibiotic prophylaxis 3-5 days preoperatively; however, this should be based on microbiological analysis to avoid unnecessary antibiotic use and reduce the risk of bacterial resistance [28,29].

Proper draping and ocular surface preparation

**Figure 5:**
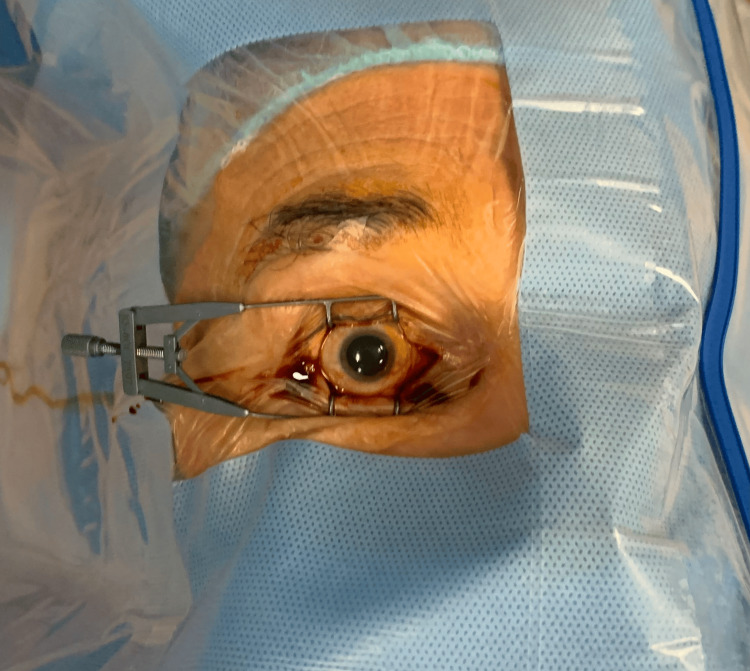
Surgical field after disinfection The skin is prepared with 10% PI and the ocular surface with 5% PI. A sterile drape and speculum have been applied. Eyelashes are isolated using surgical foil. Intraoperative picture taken by Małgorzata Ozimek, MD, PhD, in Prof. Zagorski Eye Surgery Centre in Nowy Sącz PI: povidone-iodine

After applying PI, a sterile drape with plastic foil should be placed over the surgical field. The first application of 5% PI drops should be instilled onto the ocular surface before draping (Figure 5). A second application (2 mL) should be performed after the drape and speculum are in place as a lavage over the entire ocular surface. If irrigation with normal saline or Ringer's solution is performed, PI should remain in contact with the ocular surface for at least three minutes to be effective [29]. Table 3 depicts the extempore preparation of the PI solution ready to use on the ocular surface. The ready-to-use drops are either not always available or too costly.

**Table 3:** Preparation of 5% povidone-iodine (PI) solution Table created by Aleksandra Kardaszewska, MD, based on standard operating procedures in Prof. Zagorski Eye Surgery Centre in Nowy Sącz, OCHO Medical Group BSS: balanced salt solution

Initial povidone-iodine concentration	Dilution ratio	Preparation method (10 mL syringe)
10% povidone-iodine	1:1	5 mL of 10% PI + 5 mL Ringer's solution or BSS
7.5% povidone-iodine	2:1	6 mL of 7.5% PI + 3 mL Ringer's solution or BSS

If a monocular patient wears a prosthesis, it must be removed 3-5 days preoperatively and the conjunctival sac sterilized for 3-5 days using 5% PI or other antiseptic agents [1,30].

Intraoperative endophthalmitis prophylaxis

The use of intracameral antibiotics, such as cefuroxime (Aprokam, 0.1 mg/0.1 mL) or moxifloxacin (Vigamox, off-label in Europe but approved in India as Auromox 0.5%), is essential for reducing the risk of postoperative endophthalmitis. Clinical studies show that intracameral cefuroxime can reduce infection rates from 0.59% to 0.039%, while moxifloxacin has been associated with a fourfold risk reduction in cataract patients [7,31-33]. In some centers, dropless cataract surgery involves injecting antibiotics directly into the vitreous at the end of surgery [34]. Preparations such as Tri-Moxi (triamcinolone + moxifloxacin) have been used via pars plana or transzonular routes, eliminating the need for postoperative topical drops [34]. However, combinations such as Tri-Moxi-Vanc (triamcinolone, moxifloxacin, and vancomycin) have been linked to rare but serious complications, including hemorrhagic occlusive retinal vasculitis, and are therefore not recommended [35]. As a final antiseptic measure, 5% PI should be applied to the ocular surface after wound closure and before any other drops or ointments [26].

Postoperative management

It is recommended to prescribe a high-concentration antibiotic for a short duration [8]. In practice, a combination antibiotic-steroid drop should be used four to five times daily for seven days, followed by a steroid-only drop for an additional seven days [36]. Nonsteroidal anti-inflammatory drugs (NSAIDs) are advisable twice to four times daily for 2-4 weeks postoperatively. There is no need for gradual steroid tapering [28]. The proposed postoperative treatment regimens are detailed in Table 4.

**Table 4:** Proposed postoperative and anti-inflammatory topical medication regimens following cataract surgery and combined procedures involving cataract extraction with PPV or MIGS*** Table created by Aleksandra Kardaszewska, MD *Examples: topical fluoroquinolones (levofloxacin or ofloxacin) or aminoglycosides (tobramycin or gentamycin) [9] **May be given as a combination (e.g., Ducressa, Maxitrol, Tobradex, or Dexamytrex) ***Source: [36] NSAID, nonsteroidal anti-inflammatory drug; PPV, pars plana vitrectomy; MIGS, minimally invasive glaucoma surgery

Medication (topical formulae)	Duration	Frequency of application	References
Broad-spectrum antibiotic*	5-7 days	4-5 times a day	[9,28,36]
Steroid**	2-3 weeks	4 times a day	[36]
NSAID	4 weeks if applicable	2-4 times a day	[36]

Special considerations for intravitreal injections (IVIs)

Intravitreal injections (IVIs) are performed millions of times annually for conditions such as age-related macular degeneration (AMD), diabetic macular edema (DME), or edema following retinal vein occlusion (RVO). Despite their routine nature, improper technique can result in severe complications, including clustered endophthalmitis outbreaks [4,37]. It is recommended to perform IVIs either in the operating theater or in a dedicated treatment room. All staff should wear appropriate attire and surgical masks covering the nose and mouth. Strict adherence to face masks and "no talking" policies are crucial, as studies have demonstrated that properly worn masks and "no talking" policies are both effective in preventing oral flora-associated infections [37]. The circulating nurse should prepare medications for injections in a sterile manner. If possible, prefilled syringes should be used [38]. Patients should be encouraged to maintain eyelid hygiene at home, particularly those undergoing frequent injections or monocular ones [21]. Povidone-iodine protocols for IVIs are as follows: 7.5%-10% PI applied to skin before draping and 5% PI for ocular surface before and after draping, immediately before injection, and again post-injection before drape removal [37]. The usage of PI without soap (e.g., glycerol-free) to prevent the disruption of the tear film's mucin layer is recommended. Consider using a surgical microscope or magnifying lamp for improved precision and visibility during injections [39].

Managing patients with povidone-iodine (PI) sensitivity

A true allergy to PI, i.e., an IgE-mediated (type I) hypersensitivity reaction, is rare. Most adverse reactions involve local irritation, including itching, excessive tearing, or conjunctival redness [40]. If a patient has a history of iodine sensitivity, PI can still be used at its standard concentration but should be thoroughly rinsed with balanced salt solution (BSS) immediately after application. The patient should be observed for 30-60 minutes post-procedure. If signs of intolerance occur (e.g., swelling, itching, and redness), a short course of systemic antihistamines (e.g., cetirizine or bilastine) and mild topical steroids may be prescribed for 5-7 days. In patients with confirmed PI intolerance, chlorhexidine (CHX) can be used as an alternative for ocular and skin disinfection [41]; 0.05% aqueous chlorhexidine is safe for the ocular surface [41]. Recent studies suggest that CHX is comparable to PI in preventing post-injection endophthalmitis [42-44]. While CHX may be better tolerated, it is less effective against spores and certain fungal species, making PI the gold standard unless contraindicated [22].

Antibiotics following intravitreal injections: To prescribe or not

Evidence-based medicine (EBM) does not support routine antibiotic prescription after anti-vascular endothelial growth factor (VEGF) injections [39,45,46]. Studies have shown that topical antibiotics do not reduce the rate of endophthalmitis but do contribute to bacterial resistance [45,46]. Doctors may consider antiseptic drops (e.g., containing chlorhexidine or polyhexanide) for high-risk patients, such as monocular or immunocompromised individuals, or those with severe ocular surface disease [46,47]. An exception to these recommendations is dexamethasone implant injection, where broad-spectrum antibiotics should be prescribed for 5-7 days post-injection. This is due to the larger (19 g) needle used for drug delivery, increasing the risk of bacterial contamination. However, since dexamethasone implants are administered only every 4-6 months, rather than as frequently as anti-VEGF agents, antibiotic prophylaxis remains justified [46].

By implementing these comprehensive intraoperative and postoperative protocols, the incidence of endophthalmitis can be substantially reduced both in eye surgery and IVIs, improving patient safety and surgical outcomes.

Review limitations and future directions

Although this narrative review integrates current best practices and practical guidance for the management and prevention of endophthalmitis, several limitations must be acknowledged. First, the CEVE protocol, while supported by emerging evidence and international case series, lacks large-scale randomized controlled trials directly comparing it with other strategies such as tap and inject or delayed vitrectomy. Much of the current support comes from retrospective or observational studies, which are inherently limited by selection bias and heterogeneity in practice patterns [3]. But the design and conduction of prospective randomized studies on a large scale seem to be challenging and difficult to convey nowadays, especially when the condition is quite rare and devastating. Second, while the use of povidone-iodine is universally endorsed, questions remain regarding the comparative efficacy and tolerability of alternatives such as chlorhexidine, particularly in long-term or repeated procedures such as intravitreal injections. The data here are still evolving and are often derived from smaller patient cohorts [43,48-50]. Third, despite a structured literature search covering 1995-2025 and including 50 relevant articles, this work remains a narrative review. Quantitative synthesis (meta-analysis) was not feasible, and some relevant studies may not have been captured due to language restrictions or publication bias. Fourth, there is limited discussion in the literature regarding resource-constrained environments, where immediate access to vitreoretinal surgery may not be feasible. Thus, the practical applicability of CEVE in these settings remains uncertain and warrants adaptation or triage protocols. In addition, further investigation into antiseptic alternatives should be conducted for patients with PI intolerance. These alternatives must present long-term safety for the ocular surface. Finally, the development of cost-effective, scalable protocols for low-resource settings, including optimized referral networks for surgical escalation, should be developed. The exploration of sensitive molecular diagnostic tools for the rapid identification of pathogens in endophthalmitis is necessary to better tailor therapy and avoid empirical overtreatment.

## Conclusions

The Complete Early Vitrectomy for Endophthalmitis (CEVE) strategy seems to be the most effective treatment for endophthalmitis, regardless of its underlying cause. By adhering to comprehensive infection control measures, including rapid surgical intervention, optimized prophylactic regimens, and strict perioperative protocols, ophthalmic surgeons can substantially reduce the risk of endophthalmitis and improve patient outcomes.

The use of antibiotics for endophthalmitis prevention should be limited to a high concentration for a short duration when administered as eye drops. However, intracameral antibiotics are strongly recommended at the conclusion of eye surgery, particularly following cataract extraction, phacovitrectomy, or minimally invasive glaucoma surgery (MIGS) procedures involving cataract removal. Despite ongoing controversy, we recommend against the routine use of prophylactic antibiotics following intravitreal injections, as this does not reduce the risk of endophthalmitis but contributes to bacterial resistance. Instead, proper antiseptic measures, including povidone-iodine application, remain the most reliable approach to infection prevention.
